# The impact of citrus pulp inclusion on milk performance of dairy cows: A meta-analysis

**DOI:** 10.1016/j.fochms.2024.100216

**Published:** 2024-08-13

**Authors:** Thomas Hartinger, Mubarik Mahmood, Ratchaneewan Khiaosa-ard

**Affiliations:** a Centre for Animal Nutrition and Welfare, University of Veterinary Medicine Vienna, Veterinärplatz 1, Vienna 1210, Austria; b Department of Animal Sciences, University of Veterinary and Animal Sciences, Lahore, Subcampus Jhang, Jhang 35200, Pakistan

**Keywords:** By-product, Feeding, Food waste, Milk composition, Ruminant

## Abstract

Considering sustainability and circular economy, citrus pulp could become a common dairy feedstuff. Yet, there is no clear indication of the amount of citrus pulp that can be fed without compromising milk performance or that could deliver benefits. In our meta-analysis, we investigated the impact of varying citrus pulp inclusion levels, i.e., no (0 %), low (>0–10 %), medium (>10–20 %), and high (>20 %), on milk performance variables. Replacing cereal grains with citrus pulp increased pectins and sugars while decreasing starch. At the low inclusion level, citrus pulp increased milk yield without affecting feed intake. At higher inclusion levels, citrus pulp reduced feed intake and milk yield but with higher fat contents, the milk energy content was similar to no inclusion. Concluding, citrus pulp is a good energy source with lipogenic properties in dairy cows. We encourage research to fill in the existing gap of knowledge at the rumen and systemic levels.

## Highlights

• Citrus pulp is rich in fibers, pectins and sugars but low in starch.
• Including up to 10% of citrus pulp in dairy rations improved milk yield and fat yield.
• Higher inclusion levels could affect intake but still maintain milk energy output.
• Citrus pulp has lipogenic properties by increasing milk fat precursors in the rumen.
• In support of sustainable farming, citrus pulp could become a common dairy feedstuff.

## 1 Introduction

The processing of citrus fruits for human nutrition causes high amounts of waste materials (Yadav et al., 2022), such as citrus pulp, which is further promoted by the aspiration for a healthy lifestyle and the consumers’ interest in exotic flavors (Štrbac & Savić, 2010). However, instead of discarding them as waste, such reputed agro-industrial wastes can actually be used as valuable feed for livestock and ruminants are predestinated due to their ability to efficiently utilize fibrous materials and better handle various anti-nutritive components than monogastrics (Hoffmann et al., 2003, Tripathi and Mishra, 2007).

Lactating dairy cows have high requirements for dietary energy and thus, apart from forages, starchy concentrates are typically recommended as components in dairy diets, such as corn, barley, and wheat. However, many countries face the challenge of deficits in the procurement of such grains for livestock, predominantly due to a comparably low economic status and restrictions brought by climate, technology, or infrastructure. Similarly, the dairy sector of developed countries seeks opportunities to substitute grains to reduce costs as well as increase the sustainability of milk production by mitigating feed-food competition between animal and human nutrition (Flachowsky et al., 2018).

In this context, citrus pulp may indeed represent an interesting component in dairy cow feeding. Citrus pulp comes at lower costs compared to grains and comprises large quantities of pectins and sugars, both being highly digestible for ruminants (Van Soest, 1994), as well as bioactive compounds that can exert health benefits (Schieber et al., 2001), such as a decreased systemic inflammation due to citrus flavonoids (Zhao et al., 2023). Still, the first prerequisite for using citrus pulp as a feed for dairy cows, and especially as a substitute for grains, is that the milk performance is not compromised by its inclusion in the diet. This, however, may be the case due to shifts in the diet’s carbohydrate composition influencing the proportions of rumen fermentation products that are subsequently utilized by the host for milk production. Acetate and butyrate proportions commonly increase at the expense of propionate in the rumen due to the microbial fermentation of pectins and sugars compared with starch (Hindrichsen et al., 2004, Suárez et al., 2006). This phenomenon has been also reported when substituting grains with citrus pulp (Broderick et al., 2002, Martins et al., 2021). Furthermore, it also has to be considered that components rich in pectins have a high water-holding and swelling capacity (Brachet et al., 2015) and therefore can limit feed intake due to swelling in the rumen (Voelker & Allen, 2003), which was also reported for citrus pulp (Bampidis & Robinson, 2006).

To date, several experiments have been conducted assessing the impact of citrus pulp inclusion on milk performance and describing beneficial effects or not (e.g. Williams et al., 2018, Ítavo et al., 2020). However, individually, studies hold a very limited number of inclusion levels to be tested plus the heterogeneity in basal rations and cows used among studies. Therefore, a potential dose-dependent effect of citrus pulp feeding on milk performance was not yet determined. Consequently, we aimed to generate this lacking knowledge by performing a first meta-analysis that assesses the impact of varying citrus pulp inclusion levels on the milk performance of dairy cows and so provides a first orientation on this topic. Since milk yield and components depend on rumen fermentation, we also targeted to understand its effect on rumen fermentation variables.

## 2 Materials and methods

### 2.1 Creation of the database and data management

The data for the present meta-analysis were obtained from original publications available in the Web of Science database in February 2023. For the search, keywords “cow”, “citrus pulp” and “fruit waste” were included in the topic category. Review articles, non-English articles or articles where only the abstract is accessible were excluded, meaning English full research articles were considered only without restrictions in terms of publication time, as outlined in our PRISMA flow chart (Supplementary Figure 1 according to Page et al. (2021). Experiments published within the same study were treated individually; therefore, a starting database of 30 publications with 32 experiments comprising 114 dietary treatments was created. Variables essential for the present meta-analysis included data on replicates per treatment (n), body weight (BW) of cows (kg), daily dry matter intake (DMI; kg/d), diet components (% DM), chemical composition of diets (% DM), daily milk yield (kg/d), milk protein concentration (%), milk fat concentration (%), milk lactose concentration (%), and milk urea nitrogen concentration (mg/dl). From the collected data, the ratio of milk fat to milk protein was calculated, as well as energy-corrected milk (ECM) yield (kg/d) according to Sjaunja et al. (1990) using daily milk fat yield (g/d) and daily milk protein yield (g/d). The citrus pulp intake (kg/d) was calculated from DMI and citrus pulp inclusion level. To account for different experimental and feeding plans as well as the animals used among studies, citrus pulp intake (kg/d) was further standardized by DMI and both citrus pulp intake and DMI were also related to BW (g/kg) and metabolic BW (BW^0.75^; g/kg). Similarly, ECM yield was also related to DMI (kg/kg) and BW^0.75^ (kg/kg). For the diet composition data, the concentration of pectin plus sugars (% DM) was estimated as DM (%) minus ash (% DM) minus crude protein (% DM) minus neutral detergent fiber (% DM) minus starch (% DM). For the same variables reported in different units by the studies, all data were converted into the same units of measurement.

Some of the recruited studies in the starting database did not report accurate DMI or involved dominant factors that can confound milk performance (e.g., atypical forage source or use of feed additives). We therefore managed the starting database. Specifically, the database was purged by removing experiments without information on citrus pulp inclusion level in the diet or the use of different forages between control and treatment diets. Further, experiments where citrus pulp was not offered via the diet but infused via the rumen cannula were also excluded. Pasture experiments were removed as no sufficient information on DMI could be provided, which, however, is an important influencing factor for milk performance. Similarly, the experiment using fresh sugarcane as the sole forage component was removed as it was marked an outlier when plotting DMI against ECM yield. Lastly, treatments including confounding factors besides the citrus pulp inclusion, such as probiotic feed additives or large amounts of oil and buffer compounds, were excluded from the database. Consequently, the final database used for the *meta*-analysis comprised 19 studies with 21 experiments and 59 treatments (Supplementary Table 1). The descriptive statistics of the variables are given in Supplementary Table 2.

For the final database, citrus pulp inclusion levels in the diet were additionally categorized into four categories, i.e., no, low, medium, and high that corresponded to 0 % (n = 19), >0–10 % (n = 14, inclusion level range: 2.2 – 9.8 %), >10–20 % (n = 15, inclusion level range: 11.9 19.6 %), and > 20 % (n = 11, inclusion level range: 20.0 – 56.25 %) inclusion on a DM basis, respectively. Likewise, daily citrus pulp intake was categorized into no, low, medium, and high, which corresponded to 0, >0–2, >2–4, and > 4 kg/d. Besides, the main forage species (corn vs. alfalfa) and forage type (hay vs. silage) in each treatment were determined as forage species or type that amounted to > 50 % of all forage species or types in the diet.

It is well established that diet composition affects milk yield and milk composition (NRC, 1988), such as the often observed decrease in milk fat with grain-rich feeding compared to forage-rich feeding. Therefore, in addition to the impact of citrus pulp feeding on milk performance, we also investigated rumen fermentation variables from the recruited studies, which led to 5 eligible studies. We further searched the Web of Science database for more studies investigating the impact of citrus pulp inclusion on rumen fermentation by using keyword combinations in the topic category (e.g., “citrus pulp” and “rumen”, “citrus pulp” and “ruminal fermentation”). There were 4 studied eligible, resulting in a total number of 9 experiments. The available data was not sufficient for conducting a meta-analysis and we could only compile the descriptive data for ruminal fermentation variables.

We also collected data on the chemical composition of citrus pulp reported by the retrieved studies even when it was conducted in ruminant species other than dairy cows due to a generally small number of studies available. Altogether, there were 14 studies (11 in dairy cows, 1 study in beef cattle, 1 in ewe and 1 in buffalo). However, these studies did not always report the same set of variables and thus, the number of data per nutrient variable ranged from n = 2 to n = 14. We therefore could provide only the descriptive characteristics of the chemical composition data that is presented in Table 1.

**Table 1:** Chemical composition of citrus pulp (% of dry matter unless not otherwise stated).

Type^1^	Item	n	Mean	SD	Min	Max	Median
All	Organic matter	12	92.8	1.69	90.1	95.5	93.2
	Ash	12	7.2	1.69	4.5	10.0	6.8
	Crude protein	14	7.2	1.87	4.7	12.2	7.0
	Ether extract	9	2.4	1.02	1.1	4.7	2.2
	NDF^2^	14	24.6	3.92	19.1	30.8	24.2
	ADF^3^	12	19.6	3.29	14.6	25.7	19.3
	ADL^4^	7	2.4	1.25	0.8	4.5	2.6
	Starch	6	8.6	7.01	2.2	20.3	5.3
	NFC^5^	9	57.3	6.85	47.8	67.7	57.7
	ME^6^ (MJ/kg)	3	12.8	0.39	12.5	13.2	12.6

Dried	Organic matter	9	93.2	1.57	90.1	95.5	93.2
	Ash	9	6.9	1.57	4.5	10.0	6.8
	Crude protein	10	7.1	1.95	4.8	12.2	6.9
	Ether extract	6	2.6	1.12	1.7	4.7	2.2
	NDF	10	23.4	3.49	19.1	28.6	23.7
	ADF	8	19.8	3.86	14.6	25.7	20.1
	ADL	4	2.5	1.67	0.8	4.5	2.4
	Starch	4	8.9	7.65	4.5	20.3	5.3
	NFC	6	58.9	7.80	47.8	67.7	60.4
	ME (MJ/kg)	2	12.8	0.52	12.5	13.2	12.6

^1^The category ‘All’ includes fresh, ensiled and dried forms and the category ‘Dried’ includes only dried citrus pulp as fed. ^2^Neutral detergent fiber; ^3^Acid detergent fiber; ^4^Acid detergent lignin; ^5^Non-fiber carbohydrates; ^6^Metabolizable energy.

### 2.2 Statistical analysis

All statistical analyses were performed in SAS v9.4 (SAS Institute Inc., Cary, USA). In accordance with St-Pierre (2001), the dataset was analyzed using PROC MIXED with the model (1):

(1) Y_ijklm_ = µ + C_i_ + P_j_ + S_k_ + T_l_ + (C×T)_ik_ + (C×P)_ij_ + (P×T)_jl_ + R_m_ + e_ijklm_

Where Y_ijklm_ is the dependent variable, µ is the overall mean, C_i_ is the fixed factor of citrus pulp inclusion level, P_j_ is the fixed factor of citrus pulp conservation form, S_k_ is the fixed factor of forage species, T_l_ is the fixed factor of forage type, R_m_ is the random factor of experiment and e_ijklm_ is the unexplained residual error. The interactions of forage species with other fixed factors could not be included in the model as there were no observations in each category available. Furthermore, the mixed model was weighted by replicates per treatment (Jayanegara et al., 2012). The fixed factor of daily citrus pulp intake was not additionally considered since this variable is highly correlated with the citrus pulp inclusion level in the diet, which is more standardized and not potentially confounded by the sorting behavior of cows.

Subsequently, according to St-Pierre (2001), a backward elimination method was applied using a significance value of P<0.05, meaning the stepwise removal of non-significant interactions or fixed factors from the model (1). This procedure resulted in the final model (2):

(2) Y_ij_ = µ + C_i_ + R_j_ + e_ij_

Where Y_ij_ is the dependent variable, µ is the overall mean, C_i_ is the fixed factor of citrus pulp inclusion level, R_j_ is the random factor of the experiment and e_ij_ is the unexplained residual error. Consequently, the effect of citrus pulp inclusion level as a discrete factor on the chemical composition of diet, feed intake, and milk performance was analyzed. The significance level was set at P≤0.05 and a trend was defined as 0.05 < P<0.10.

## 3 Results

### 3.1 Chemical composition of citrus pulp products

The chemical composition of all citrus pulp products is presented in Table 1. The data revealed only marginal differences in nutrient and energy concentration when considering dried citrus pulp only or all types of citrus pulp, meaning fresh, ensiled, and dried. With a median of 5.3 % of DM, the citrus pulp products were low in starch, except for one dried sample containing 20.3 % starch in DM. Likewise, crude protein and ether extract showed medians of 7.0 % and 2.2 % of DM, respectively, and amounted together for less than 10 % of citrus pulp. Therefore, non-fiber carbohydrates, i.e., starch, sugars, and pectins, constituted the largest fraction in citrus pulp products, amounting to on average nearly 60 % of DM. Since the mean starch concentration was 8.6 % in DM, the majority of the non-fiber carbohydrate fraction constituted of sugars and pectins.

### 3.2 Effect of citrus pulp inclusion on diet composition and feed intake

The results for the effect of citrus pulp inclusion on diet composition and feed intake are provided in Table 2. The analysis showed that ash concentration in the diet was higher with low and medium citrus pulp inclusion compared to no inclusion, while the medium inclusion level did not differ from other categories (P=0.05). The starch level continuously decreased with higher inclusion of citrus pulp, leading to the highest values for no inclusion and the lowest for high citrus pulp inclusion (P<0.01). Likewise, the estimated contents of pectin plus sugars followed a reverse pattern with the highest values for high inclusion and the lowest for no inclusion (P<0.01). Regarding fiber components, acid detergent lignin concentrations were higher with the inclusion of citrus pulp than without, irrespective of inclusion amount (P=0.01). Moreover, a trend for more neutral detergent fiber with the medium inclusion level than for no inclusion was observed (P=0.06). The inclusion of citrus pulp showed no effect on the concentrations of DM, organic matter, crude protein, and ether extract (each P>0.10).

**Table 2:** Effect of citrus pulp inclusion level on diet composition and feed intake of dairy cows.

	Inclusion level^1^					
Variable	No	Low	Medium	High	SEM^2^	P-value
DM^3^ concentration (%)	46.8	45.1	46.5	45.1	3.13	0.39
Ash (% DM)	7.58^b^	9.30^a^	8.60^ab^	9.84^a^	1.48	0.05
Organic matter (% DM)	93.0	91.6	92.0	90.4	1.87	0.14
Crude protein (% DM)	17.2	17.0	17.1	17.2	0.55	0.95
Ether extract (% DM)	3.46	4.04	3.49	3.64	0.39	0.37
NDF^4^ (% DM)	31.4^y^	33.0^xy^	33.7^x^	33.2^xy^	1.31	0.06
ADF^5^ (% DM)	18.8^b^	22.3^a^	21.9^a^	21.1^a^	1.93	0.01
Starch (% DM)	31.8^a^	22.1^b^	21.2^b^	15.6^c^	1.89	<0.01
Pectin plus sugars^6^ (% DM)	13.3^c^	17.2^b^	20.2^ab^	22.6^a^	1.72	<0.01
DMI^7^ (kg/d)	20.3^ab^	20.5^a^	19.3^c^	19.7^bc^	0.78	<0.01
DMI (g/kg BW^8^)	34.6^a^	34.7^ab^	30.7^b^	32.9^ab^	1.67	0.04
DMI (g/kg BW^0.75^)	165^ab^	168^a^	157^c^	159^bc^	7.59	<0.01
Citrus pulp intake (kg DM/d)	0.00^d^	1.70^c^	2.94^b^	4.29^a^	0.16	<0.01
Citrus pulp intake (g/kg BW)	0.00^d^	2.76^c^	4.90^b^	8.18^a^	0.37	<0.01
Citrus pulp intake (g/kg BW^0.75^)	0.00^d^	13.6^c^	24.4^b^	40.4^a^	1.84	<0.01

^1^No = 0 % inclusion, Low = >0–10 % inclusion, Medium = >10–20 % inclusion, High = >20 % inclusion (% of diet dry matter); ^2^Standard error of the mean; ^3^Dry matter; ^4^Neutral detergent fiber; ^5^Acid detergent fiber; ^6^Estimated as dry matter − ash − crude protein − neutral detergent fiber – starch; ^7^Dry matter intake; ^8^Body weight.

Numbers with different superscript letters within a row indicate significant differences (P<0.05).

The daily DMI was highest with low citrus pulp inclusion and higher than medium and high inclusion, but not differing from no inclusion, while medium inclusion level resulted in a lower daily DMI than with no inclusion of citrus pulp (P<0.01). The same pattern was also true for DMI related to BW^0.75^ (P<0.01). When relating DMI to BW, numbers for no inclusion were higher than for medium inclusion, whereas the other categories did not differ (P=0.04). Regarding citrus pulp intake, a continuous increase was present from no inclusion to high inclusion and this was consistent for daily citrus pulp intake as well as citrus pulp intake related to BW or BW^0.75^ (each P<0.01).

### 3.3 Effect of citrus pulp inclusion on milk performance

The results for milk yield and milk components are presented in Table 3. The daily milk yield was higher with low inclusion than the other categories, while no inclusion of citrus pulp led to a higher daily milk yield compared to the high inclusion level (P<0.01). For the ECM yield, low inclusion of citrus pulp again had the highest numbers, which were higher than all other categories that did not differ from each other (P<0.01). When ECM yield was related to DMI, treatments with no inclusion of citrus pulp showed lowest numbers that were lower than with high and even more than with low inclusion (P=0.01).

**Table 3:** Effect of citrus pulp inclusion level on milk yield and composition in dairy cows.

	Inclusion level^1^					
Variable	No	Low	Medium	High	SEM^2^	P-value
Milk yield (kg/d)	29.0^b^	30.9^a^	28.4^bc^	28.0^c^	1.23	<0.01
ECM^3^ yield (kg/d)	26.2^b^	28.2^a^	25.7^b^	26.2^b^	1.05	<0.01
ECM yield (kg/kg DMI^4^)	1.30^c^	1.42^a^	1.33^bc^	1.38^ab^	0.05	0.01
ECM yield (kg/kg BW^0.75^)	0.220	0.234	0.217	0.226	0.01	0.56
Protein (%)	3.09	3.08	3.01	3.12	0.07	0.22
Fat (%)	3.37^b^	3.44^b^	3.41^b^	3.63^a^	0.10	<0.01
Fat protein ratio	1.09^b^	1.12^b^	1.14^ab^	1.17^a^	0.04	0.04
Lactose (%)	4.71^a^	4.65^ab^	4.64^b^	4.62^b^	0.06	0.01
Milk urea nitrogen (mg/dl)	15.0	13.7	14.5	14.0	1.26	0.85
Protein yield (g/d)	891^b^	947^a^	852^c^	862^bc^	34.4	<0.01
Fat yield (g/d)	969^b^	1062^a^	961^b^	1012^b^	44.3	0.03
Lactose yield (g/d)	1328	1370	1252	1357	75.3	0.13

^1^No = 0 % inclusion, Low = >0–10 % inclusion, Medium = >10–20 % inclusion, High = >20 % inclusion (% of diet dry matter); ^2^Standard error of the mean; ^3^Energy-corrected milk; ^4^Dry matter intake.

Numbers with different superscript letters within a row indicate significant differences (P<0.05).

Regarding milk composition, high citrus pulp inclusion resulted in up to 0.26 percentage points higher milk fat concentrations than for other categories that were similar (P<0.01). Similarly, the milk fat to milk protein ratio was higher for high inclusion than for no and low inclusion with medium inclusion as intermediate (P=0.04). The lactose concentration, however, was higher without citrus pulp than with medium or high inclusion, while low inclusion did not differ (P=0.01).

Both protein and fat yield were highest in studies with low citrus pulp inclusion and higher than all other categories (P<0.01 and P=0.03 for protein and fat yield, respectively). For protein yield only, the category of no inclusion had higher yields than the medium inclusion category. The inclusion of citrus pulp showed no effect on the ECM yield related to BW^0.75^ or on concentrations of milk protein and milk urea nitrogen as well as lactose yield (each P>0.10).

### 3.4 Effect of citrus pulp inclusion on rumen fermentation

The influences of citrus pulp inclusion on main rumen fermentation variables are summarized descriptively in Table 4 and the majority of studies were conducted with Holstein cows fed corn silage-based diets. Most studies observed no changes in ruminal pH with citrus pulp inclusion, while the ammonia concentration commonly declined. Apart from one study, short-chain fatty acid profiles in the rumen were altered in response to citrus pulp feeding: Increments in acetate and butyrate proportions were accompanied by reductions of the branched-chain fatty acids *iso*-butyrate and *iso*-valerate. In contrast, the propionate proportion was often decreased with higher citrus pulp inclusion, whereas total short-chain fatty acid concentrations followed no clear pattern and were partly unaffected, increased, or decreased.

**Table 4:** Descriptive effects of citrus pulp inclusion on rumen fermentation variables.

#	Main experimental characteristics	Effects with higher citrus pulp inclusion			Reference
		pH	Short-chain fatty acids (SCFA)	Ammonia	
1*	• 11 Holstein dairy cows • Ad libitum feeding of an isonitrogenous, corn silage-based total mixed ration (TMR) for 21 days • Low vs. high citrus pulp inclusion (2.2 vs. 23.6 % of DM^1^)	–^2^	−	−	(Leiva et al., 2000)
2*	• 6 Holstein dairy cows • Ad libitum feeding of an isonitrogenous, alfalfa silage-based TMR for 21 days • No vs. medium citrus pulp inclusion (0.0 vs. 19.1 % of DM)	↓^3^	Total SCFA ↑^4^ Propionate ↓ Butyrate↑ Valerate ↑ Iso-butyrate ↓ Iso-valerate ↓	↓	(Broderick et al., 2002)
5*	• 32 Holstein dairy cows • Restricted feeding of alfalfa cubes (14.5 kg DM/d) and concentrate mix (6 kg DM/d) for 21 days • No vs. medium citrus pulp inclusion (0.0 vs. 13.2 % of DM)	−	Iso-butyrate ↓ Caproate ↑	↓	(Williams et al., 2018)
6*	• 16 Holstein dairy cows • Ad libitum feeding of a corn silage-based TMR for 21 days • No vs. high citrus pulp inclusion (0.0 vs. 26.2 % of DM)	−	Acetate ↑ Propionate ↓ Butyrate↑ Iso-butyrate ↓ Iso-valerate ↓ Acetate/propionate ratio ↑	↓	(Martins et al., 2021)
7	• 6 Jersey dairy cows • Ryegrass pasture with concentrate mix (6 kg DM/d) for 51 days • No vs. medium citrus pulp inclusion (0.0 vs. 13.8 % of DM)	−	Iso-butyrate ↓ Iso-valerate ↓	↑	(Steyn et al., 2017)
8	• 8 Holstein dairy cows • Ad libitum feeding of an isonitrogenous, corn silage-based TMR or citrus peel-silage-based TMR for 21 days • No vs. medium vs. high citrus pulp inclusion (0.0 vs. 18.8 % vs. 37.5 vs. 56.3 of DM)	↑	Total SCFA ↓	↑	(Ítavo et al., 2020)
9*	• 4 Holstein dairy cows • Ad libitum feeding of an isonitrogenous, corn silage-based TMR for 21 days • Low vs. high citrus pulp inclusion (9.7 vs. 23.9 % of DM)	−	Not analyzed	Not analyzed	(Solomon et al., 2000)

^1^Dry matter; ^2^No difference between varying citrus pulp inclusion rates; ^3^Reduction compared to control or lower citrus pulp inclusion; ^4^Increase compared to control or lower citrus pulp inclusion; *Studies included in the database for analysis of intake and milk performance.

## 4 Discussion

There is a rapidly growing interest in feed sources alternative to cereal grains for decreasing feed-food competition and costs as well as optimizing rumen health. In this context, citrus pulp represents an attractive feed source for ruminants because of its high energy content (12.8 ± 0.39 MJ metabolizable energy/kg DM in our meta-analysis) while still providing more structural carbohydrates (24.6 ± 3.92 % neutral detergent fiber in DM in our meta-analysis) than typically achieved by cereal grains (Jeroch et al., 2020). However, substitution of starchy grains by the non-forage fiber source citrus pulp means a considerable shift in the carbohydrate fraction, i.e., a decline in starch vs. rise in pectins and sugars, as well as the threat of a reduced feed intake due to swelling in the rumen (Bampidis & Robinson, 2006). Both aspects may eventually affect the milk performance of dairy cows, which has not yet been comprehensively assessed and therefore was the objective of the present study.

Our data clearly showed a dose-dependent effect of citrus pulp inclusion on feed intake and milk performance of dairy cows. The low inclusion level, i.e., below 10 % of diet on a DM basis, led to the highest DMI of all categories and was also higher than no inclusion, which may be related to increased palatability of the diet with citrus pulp by introducing additional flavors (Castillo-Lopez et al., 2021). When fed in higher amounts, bitter compounds, such as limonin, can then compromise acceptance (Bampidis & Robinson, 2006) as we correspondingly observed for DMI at medium and high inclusion levels in our meta-analysis. In addition to the affected palatability, with the physical property of citrus pulp to swell with hydration (Brachet et al., 2015), thereby promoting the rumen fill but limiting feed intake (Bampidis & Robinson, 2006), it is likely that elevated inclusion levels of dried citrus pulp (>10 % of diet on a DM basis) at the expense of starchy grains can negatively affect intake.

Interestingly, although feed intake was not different between no and low citrus pulp inclusion, milk yield was significantly higher for the low inclusion level. Therefore, other factors than solely DMI were decisive and changes in the carbohydrate composition of the diet may have contributed to the effect. It is worth of remark that the starch concentration strongly dropped by 10 percentage points from no to low inclusion, whereas the milk yield increased. The simultaneous increment of pectins and sugars with more citrus pulp inclusion seemed to overcompensate the starch decline, but only when fed below 10 % of the diet. Indeed, pectins are considered the most rapidly fermentable complex carbohydrate (Van Soest, 1994) and the commonly observed increases of acetate and butyrate proportions in the rumen at the expense of propionate support this observation (Broderick et al., 2002, Martins et al., 2021). Apart from alterations in the carbohydrate profile, the higher presence of bioactive compounds, such as phenolics and flavonoids, with the inclusion of citrus pulp might be another part of the answer, albeit related effects in literature are small and inconsistent (Bampidis and Robinson, 2006, Tayengwa and Mapiye, 2018). Therefore, this observation of higher milk yield but similar DMI for low vs. no citrus pulp inclusion demands further investigation. Additionally, we acknowledge the possibility that other factors than citrus pulp inclusion may have confounded the observed effect, which can be especially the case at the low inclusion level and despite the thorough analysis and management of the database.

When converting the milk into ECM, the low citrus pulp inclusion level again led to the highest yield among all categories, therefore demonstrating that the higher uncorrected milk yield was not due to “diluted milk”. This became also obvious since protein and fat yields were as well highest for the low inclusion level. Moreover, our findings also revealed that ECM yield for both medium and high inclusion was not different from no citrus pulp inclusion, which may be mainly ascribed to the numerically or significantly higher milk fat concentration with medium and high citrus pulp inclusion. Furthermore, this also resulted in similar fat yields for no, medium, and high citrus pulp inclusion. As ruminal proportions of acetate and butyrate typically increase with the intake of citrus pulp (Broderick et al., 2002, Martins et al., 2021), there would be more of these lipogenic precursors at the mammary gland and therefore explain this phenomenon. On the contrary, a reduction of propionate − the precursor for glucose synthesis and thus mammary lactose production − when feeding citrus pulp has also been observed (Broderick et al., 2002, Martins et al., 2021). As the main milk osmole, lactose determines the water secretion and thus milk yield (Miglior et al., 2006). In our data, medium and high citrus pulp inclusion decreased lactose percentages compared to no inclusion. Although the biological gap of around 0.1 percentage unit was small, it may already reflect lower lactose synthesis that could partly explain the decreased milk yield when feeding high levels of citrus pulp, together with the reduced DMI. However, since the ECM yield maintained similar but the DMI declined with high compared to no citrus pulp inclusion, we can actually interpret this as a higher milk production efficiency for high than for no inclusion, which is in accordance with earlier findings (Miron et al., 2002). This suggests that citrus pulp may indeed compensate for starchy supplements also at higher inclusion rates when considered relative to feed intake. Still, in absolute and relative dimensions, low citrus pulp inclusion was constantly superior to all other categories.

On a diet level, our meta-analysis further showed that the chemical composition was less influenced by citrus pulp inclusion, except for the starch and pectin plus sugars proportions. This may be explained by the fact that dairy cow diets are typically balanced for nutrients and therefore the low crude protein content of citrus pulp is counteracted by the addition of crude protein-rich feeds, such as soybean meal or other legumes. Likewise, concentrations of protein and urea nitrogen in the milk were not affected by varying citrus pulp inclusion and our findings suggest that citrus pulp feeding does not pose the general risk of nitrogen scarcity. Still, the ammonia levels in the rumen were often reported to decline with more citrus pulp in the diet (Broderick et al., 2002, Williams et al., 2018, Martins et al., 2021). As the nitrogen supply in those studies did not change, the impact of citric essential oils and other bioactive compounds may be responsible (Tayengwa & Mapiye, 2018), although as well unaffected or increased ruminal ammonia concentrations in response to citrus pulp feeding are documented (Leiva et al., 2000, Steyn et al., 2017, Ítavo et al., 2020), which should be noted here. As a side aspect, it is noteworthy that citrus pulp may be unsuitable for close-up diets due to its high Ca levels that could trigger milk fever when fed in high amounts (Bampidis & Robinson, 2006), whereas it may indeed be a valuable feed in lactation diets and only the Ca:P imbalance has to be considered. Certainly, the limited size of the present data set has to be acknowledged and further studies on citrus pulp inclusion are requested to help to substantiate our findings. Since the majority of analyzed studies fed diets with corn silage as the sole or dominant forage source, the insights of our meta-analysis may especially apply to the scenario of feeding corn silage-based diets, which indeed represents the main forage in modern dairy production systems (Adesogan et al., 2020). The absence of significant interactions between citrus pulp inclusion and forage source or forage species though indicates that the observed improvements in milk performance with low citrus pulp inclusion are rather universal, but again, further research and higher study numbers are warranted for confirmation. Likewise, the effects of grain replacement by citrus pulp might vary or be differently pronounced between different grain sources, such as corn, barley or wheat, which, however, could not yet be examined with the limited data set and should be pursued in the future.

Moreover, apart from milk yield and main milk components that were the focus of our study, research regarding the impact of citrus pulp feeding on the sensory properties of milk is advisable. Available studies analyzing the quality of raw milk cheese from goats fed up to 39 % citrus pulp (DM basis) as well as meat from steers fed up to 30 % citrus pulp (DM basis) provide no evidence for effects on these products (Guzmán et al., 2020, Luzardo et al., 2021). Similarly, Jaramillo et al. (2009) found no influence on milk and cheese quality when feeding lactating ewes up to 30 % whole citrus (DM basis). Still, potential changes in the sensory properties of milk when including larger amounts of citrus pulp in dairy diets may be kept in mind.

## 5 Conclusions

In summary, including up to 10 % citrus pulp in dairy cow diets improved milk performance as evidenced by higher yields of milk, fat, and protein, which, however, was not fully traced back to a higher DMI. At higher citrus pulp inclusion rates, DMI and milk yield were depressed, although expressed as ECM, higher inclusion rates did not result in lower performance and even suggested a more efficient transfer of energy in feed into milk. Therefore, based on our findings, citrus pulp may be used as a substitute for starchy supplements in dairy cow feeding, especially in lower quantities, i.e. up to 10 % of diet on a DM basis. From the milk data, citrus pulp represents a lipogenic feed source for dairy cows. There is a need for more insights into the effect of feeding citrus pulp on rumen microbiota as well as on the host’s systemic responses, which can benefit from a multiomics approach. Such insights are encouraged to substantiate our current findings and further the knowledge about functional and metabolic effects as well as the safety of feeding citrus pulp in dairy cows.

## Funding statement

This research is part of the project “Turning fruit and vegetable wastes into livestock feed: Sustainable feed resources with functional properties” (Project number KoEF 06/2020). The project is financed by the Austrian Federal Ministry of Education, Science and Research within the program “Cooperation Development Research” administered by OeAD GmbH Austria’s Agency for Education and Internationalisation.

## CRediT authorship contribution statement

**Thomas Hartinger:** Writing – review & editing, Writing – original draft, Visualization, Validation, Software, Methodology, Investigation, Formal analysis, Data curation. **Mubarik Mahmood:** Writing – review & editing, Investigation, Data curation. **Ratchaneewan Khiaosa-ard:** Writing – review & editing, Validation, Supervision, Project administration, Methodology, Funding acquisition, Conceptualization.

## Declaration of competing interest

The authors declare that they have no known competing financial interests or personal relationships that could have appeared to influence the work reported in this paper.

## Data Availability

The data set used for the meta-analysis is shown in Supplementary Table 1.
