# Supplementary material for: The impact of citrus pulp inclusion on milk performance of dairy cows: A meta-analysis
Source: Food Chem (Oxf). 2024 Aug 13;9:100216. doi: 10.1016/j.fochms.2024.100216 (PMC11381455; doi:10.1016/j.fochms.2024.100216)
Supplement: Supplementary Data 1 [file mmc1.docx]

**
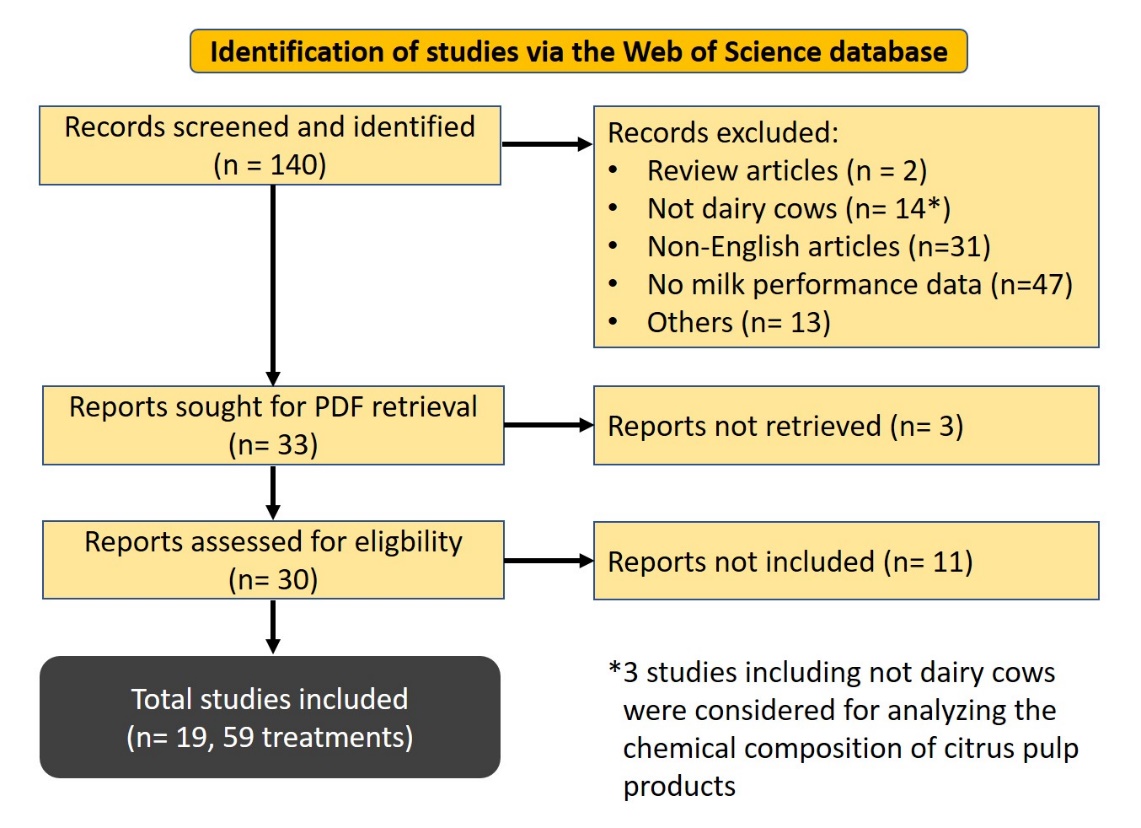
**

**Supplementary Figure 1.** PRISMA flow chart illustrating the creation of the database used for the meta-analysis.

**Supplementary Table 2.** Overview on data distribution across all studies included in the analysis.

| Variable | Inclusion level | N | Mean | SD | Minimum | Maximum |
| --- | --- | --- | --- | --- | --- | --- |
| Body weight (kg) | No | 19 | 617 | 22.86 | 586 | 650 |
|  | Low | 12 | 607 | 24.96 | 563 | 650 |
|  | Medium | 14 | 619 | 22.21 | 588 | 650 |
|  | High | 9 | 594 | 23.94 | 563 | 635 |
| Metabolic body weight (kg) | No | 19 | 124 | 3.44 | 119 | 129 |
|  | Low | 12 | 122 | 3.78 | 116 | 129 |
|  | Medium | 14 | 124 | 3.34 | 119 | 129 |
|  | High | 9 | 120 | 3.64 | 116 | 127 |
| Experiment duration (d) | No | 19 | 92 | 25.92 | 28 | 140 |
|  | Low | 14 | 73 | 26.42 | 24 | 140 |
|  | Medium | 15 | 86 | 27.56 | 28 | 140 |
|  | High | 11 | 68 | 20.22 | 24 | 84 |
| Citrus inclusion level (%) | No | 19 | 0.00 | 0.00 | 0.00 | 0.00 |
|  | Low | 14 | 7.35 | 2.73 | 2.20 | 9.80 |
|  | Medium | 15 | 15.40 | 2.82 | 11.90 | 19.60 |
|  | High | 11 | 28.91 | 13.02 | 20.00 | 56.25 |
| Forage proportion (%) | No | 19 | 54.92 | 9.12 | 40.00 | 75.00 |
|  | Low | 14 | 43.35 | 6.82 | 32.80 | 60.00 |
|  | Medium | 15 | 50.39 | 8.13 | 39.70 | 60.00 |
|  | High | 11 | 40.30 | 9.35 | 18.75 | 50.00 |
| Concentrate proportion (%)^1^ | No | 19 | 45.08 | 9.12 | 25.00 | 60.00 |
|  | Low | 14 | 56.65 | 6.82 | 40.00 | 67.20 |
|  | Medium | 15 | 49.61 | 8.13 | 40.00 | 60.30 |
|  | High | 11 | 59.70 | 9.35 | 50.00 | 81.25 |
| Dry matter intake (kg/d) | No | 19 | 20.41 | 3.14 | 10.00 | 23.40 |
|  | Low | 14 | 22.13 | 2.51 | 16.50 | 25.20 |
|  | Medium | 15 | 19.76 | 3.28 | 9.50 | 22.68 |
|  | High | 11 | 18.32 | 5.26 | 8.90 | 23.90 |
| Dry matter intake (g/kg BW^2^) | No | 19 | 33.7 | 5.97 | 20.0 | 40.0 |
|  | Low | 12 | 36.7 | 4.92 | 30.0 | 40.0 |
|  | Medium | 14 | 31.4 | 5.35 | 20.0 | 40.0 |
|  | High | 9 | 32.2 | 8.33 | 20.0 | 40.0 |
| Dry matter intake (g/kg BW^0.75^) | No | 19 | 164.7 | 25.0 | 80.0 | 190.0 |
|  | Low | 12 | 181.7 | 21.2 | 130.0 | 200.0 |
|  | Medium | 14 | 159.3 | 26.4 | 80.0 | 180.0 |
|  | High | 9 | 147.8 | 46.8 | 70.0 | 190.0 |
| Citrus pulp intake (kg/d) | No | 19 | 0.00 | 0.00 | 0.00 | 0.00 |
|  | Low | 14 | 1.60 | 0.59 | 0.47 | 2.47 |
|  | Medium | 15 | 3.00 | 0.57 | 1.78 | 3.92 |
|  | High | 11 | 4.78 | 1.06 | 3.38 | 7.35 |
| Citrus pulp intake (kg/kg BW) | No | 19 | 0.00 | 0.00 | 0.00 | 0.00 |
|  | Low | 12 | 0.00 | 0.00 | 0.00 | 0.00 |
|  | Medium | 14 | 0.00 | 0.00 | 0.00 | 0.01 |
|  | High | 9 | 0.01 | 0.00 | 0.01 | 0.01 |
| Citrus pulp intake (kg/kg BW^0.75^) | No | 19 | 0.00 | 0.00 | 0.00 | 0.00 |
|  | Low | 12 | 0.01 | 0.00 | 0.01 | 0.02 |
|  | Medium | 14 | 0.02 | 0.01 | 0.01 | 0.03 |
|  | High | 9 | 0.04 | 0.01 | 0.03 | 0.06 |
| Dry matter (%) | No | 8 | 49.95 | 7.69 | 40.00 | 58.70 |
|  | Low | 7 | 49.74 | 8.44 | 41.90 | 62.95 |
|  | Medium | 8 | 44.72 | 10.27 | 22.70 | 58.90 |
|  | High | 9 | 47.04 | 9.51 | 35.44 | 63.80 |
| Organic matter (% DM^3^) | No | 9 | 92.37 | 5.62 | 78.00 | 96.53 |
|  | Low | 12 | 92.72 | 1.36 | 90.70 | 95.20 |
|  | Medium | 10 | 93.46 | 1.23 | 91.00 | 95.66 |
|  | High | 8 | 89.71 | 10.03 | 65.00 | 94.50 |
| Ash (% DM) | No | 16 | 9.06 | 4.18 | 3.47 | 22.00 |
|  | Low | 13 | 7.71 | 1.19 | 5.60 | 9.30 |
|  | Medium | 13 | 8.04 | 2.35 | 4.34 | 11.40 |
|  | High | 10 | 10.12 | 8.85 | 5.50 | 35.00 |
| Crude protein (% DM) | No | 18 | 18.09 | 2.91 | 12.70 | 22.70 |
|  | Low | 14 | 17.04 | 0.96 | 15.50 | 19.05 |
|  | Medium | 14 | 17.67 | 2.45 | 12.80 | 22.10 |
|  | High | 11 | 16.33 | 1.98 | 13.00 | 19.15 |
| Ether extract (% DM) | No | 13 | 3.03 | 0.54 | 1.62 | 3.60 |
|  | Low | 6 | 4.75 | 1.28 | 2.91 | 6.10 |
|  | Medium | 11 | 3.27 | 0.94 | 1.79 | 5.40 |
|  | High | 5 | 3.14 | 1.56 | 1.96 | 5.80 |
| Neutral detergent fiber (% DM) | No | 18 | 29.53 | 5.29 | 24.80 | 46.10 |
|  | Low | 14 | 33.30 | 2.96 | 27.00 | 38.80 |
|  | Medium | 13 | 31.41 | 2.07 | 28.40 | 33.80 |
|  | High | 11 | 34.06 | 5.43 | 28.00 | 47.80 |
| Acid detergent fiber (% DM) | No | 10 | 17.66 | 5.38 | 12.50 | 31.00 |
|  | Low | 5 | 20.72 | 2.89 | 16.90 | 23.60 |
|  | Medium | 7 | 19.20 | 1.79 | 16.73 | 21.60 |
|  | High | 6 | 21.83 | 6.80 | 16.10 | 33.60 |
| Starch (% DM) | No | 12 | 31.23 | 6.80 | 23.40 | 42.30 |
|  | Low | 10 | 21.57 | 5.22 | 12.20 | 26.45 |
|  | Medium | 10 | 20.53 | 3.91 | 14.80 | 26.80 |
|  | High | 5 | 15.69 | 4.05 | 12.90 | 22.80 |
| Pectin estimation^4^ (% DM) | No | 12 | 13.78 | 1.59 | 11.60 | 16.30 |
|  | Low | 10 | 19.32 | 6.01 | 11.80 | 29.70 |
|  | Medium | 10 | 20.81 | 4.43 | 10.70 | 27.30 |
|  | High | 5 | 24.21 | 2.67 | 21.05 | 27.40 |
| Milk yield (kg/d) | No | 19 | 29.84 | 5.44 | 19.30 | 38.20 |
|  | Low | 13 | 34.06 | 5.25 | 23.80 | 41.00 |
|  | Medium | 15 | 27.99 | 4.04 | 18.60 | 36.00 |
|  | High | 10 | 29.28 | 7.74 | 17.40 | 38.60 |
| ECM^5^ yield (kg/d) | No | 19 | 27.06 | 4.42 | 17.49 | 33.72 |
|  | Low | 13 | 30.38 | 5.03 | 18.96 | 36.40 |
|  | Medium | 15 | 25.65 | 3.69 | 17.60 | 31.95 |
|  | High | 10 | 27.04 | 6.05 | 17.96 | 35.29 |
| ECM yield (kg/kg DMI^6^) | No | 19 | 1.34 | 0.17 | 1.13 | 1.75 |
|  | Low | 13 | 1.37 | 0.11 | 1.15 | 1.53 |
|  | Medium | 15 | 1.32 | 0.18 | 1.18 | 1.85 |
|  | High | 10 | 1.56 | 0.26 | 1.32 | 2.02 |
| ECM yield (kg/kg BW^0.75^) | No | 19 | 0.22 | 0.04 | 0.15 | 0.28 |
|  | Low | 11 | 0.25 | 0.05 | 0.15 | 0.30 |
|  | Medium | 14 | 0.22 | 0.03 | 0.16 | 0.26 |
|  | High | 8 | 0.24 | 0.05 | 0.15 | 0.29 |
| Protein (%) | No | 19 | 3.09 | 0.13 | 2.77 | 3.29 |
|  | Low | 13 | 2.92 | 0.18 | 2.62 | 3.13 |
|  | Medium | 15 | 3.11 | 0.29 | 2.66 | 3.74 |
|  | High | 10 | 3.01 | 0.33 | 2.64 | 3.70 |
| Fat (%) | No | 19 | 3.39 | 0.38 | 2.57 | 4.12 |
|  | Low | 13 | 3.30 | 0.28 | 2.40 | 3.47 |
|  | Medium | 15 | 3.44 | 0.39 | 2.40 | 3.94 |
|  | High | 10 | 3.65 | 0.41 | 3.09 | 4.48 |
| Fat protein ratio | No | 18 | 1.10 | 0.13 | 0.82 | 1.28 |
|  | Low | 12 | 1.14 | 0.14 | 0.77 | 1.32 |
|  | Medium | 13 | 1.11 | 0.16 | 0.75 | 1.32 |
|  | High | 10 | 1.22 | 0.13 | 1.00 | 1.39 |
| Lactose (%) | No | 14 | 4.78 | 0.15 | 4.51 | 5.05 |
|  | Low | 5 | 4.52 | 0.08 | 4.45 | 4.62 |
|  | Medium | 12 | 4.61 | 0.16 | 4.46 | 4.93 |
|  | High | 3 | 4.72 | 0.27 | 4.57 | 5.03 |
| Milk urea nitrogen (mg/dl) | No | 12 | 15.35 | 3.17 | 11.30 | 19.80 |
|  | Low | 10 | 13.95 | 2.69 | 8.81 | 18.30 |
|  | Medium | 11 | 14.80 | 3.05 | 10.50 | 20.30 |
|  | High | 5 | 12.83 | 2.25 | 9.57 | 15.70 |
| Protein yield (g/d) | No | 19 | 919 | 168 | 580 | 1190 |
|  | Low | 13 | 991 | 121 | 740 | 1150 |
|  | Medium | 15 | 869 | 135 | 540 | 1140 |
|  | High | 10 | 862 | 174 | 600 | 1110 |
| Fat yield (g/d) | No | 19 | 1002 | 164 | 660 | 1290 |
|  | Low | 13 | 1133 | 229 | 570 | 1420 |
|  | Medium | 15 | 961 | 158 | 590 | 1190 |
|  | High | 10 | 1049 | 226 | 710 | 1420 |
| Lactose yield (g/d) | No | 14 | 1400 | 220 | 1067 | 1770 |
|  | Low | 5 | 1378 | 193 | 1095 | 1640 |
|  | Medium | 12 | 1290 | 138 | 1136 | 1642 |
|  | High | 3 | 1328 | 220 | 1187 | 1581 |

^1^Citrus pulp was considered as concentrate; ^2^Body weight; ^3^Dry matter; ^4^Calculated as dry matter - ash - crude protein - neutral detergent fiber - starch; ^5^Energy-corrected milk; ^6^Dry matter intake.
